# Supplementary material for: The Simultaneous Detection of Multiple Antibiotics in Milk and Pork Based on an Antibody Chip Biosensor
Source: Biosensors (Basel). 2022 Jul 29;12(8):578. doi: 10.3390/bios12080578 (PMC9405744; doi:10.3390/bios12080578)
Supplement: Supplementary file 1 [file biosensors-12-00578-s001.zip › biosensors-1807106-supplementary.pdf]

Supplementary material

Table S1. Square matrix titration of CLX monoclonal antibody of ELISA

Mab dilution (1:X)	Coating antigen(μg/mL)							
	1	0.5	0.25	0.125	0.0625	0.03125	0.015625	0.007813
5000	3.139	3.188	2.920	1.684	0.833	0.444	0.480	0.457
10000	3.486	3.245	2.088	0.890	0.324	0.208	0.166	0.145
20000	2.958	2.152	0.694	0.227	0.135	0.104	0.099	0.102
40000	1.279	0.767	0.246	0.125	0.102	0.098	0.103	0.097
80000	0.194	0.148	0.106	0.095	0.094	0.094	0.099	0.094
160000	0.100	0.091	0.089	0.091	0.092	0.093	0.092	0.093
320000	0.090	0.090	0.086	0.090	0.089	0.091	0.091	0.090
640000	0.087	0.087	0.097	0.088	0.090	0.092	0.091	0.101

Table S2. Square matrix titration of CRO monoclonal antibody of ELISA

Coating antigen	Mab dilution (1:X)							
(μg/mL)	5000	10000	20000	40000	80000	160000	320000	640000
16	3.271	2.607	0.991	0.475	0.271	0.135	0.300	0.261
8	3.120	2.582	0.921	0.487	0.338	0.133	0.221	0.170
4	2.923	2.048	0.584	0.356	0.342	0.186	0.123	0.248
2	2.188	1.111	0.327	0.252	0.150	0.146	0.127	0.207
1	0.963	0.358	0.354	0.317	0.165	0.133	0.287	0.121
0.5	0.581	0.350	0.306	0.313	0.279	0.141	0.122	0.116

Table S3. Square matrix titration of N monoclonal antibody of ELISA

Mab dilution (1:X)	Coating antigen (μg/mL)							
	16	8	4	2	1	0.5	0.25	0.125
1000	3.279	3.021	2.774	2.791	1.945	0.867	0.660	0.488
2000	3.806	3.582	3.322	2.961	1.804	0.727	0.470	0.616
4000	3.642	3.473	3.263	2.882	1.414	0.485	0.212	0.128
8000	2.788	2.769	2.669	2.19	0.858	0.328	0.163	0.109
16000	1.419	1.436	1.453	1.182	0.521	0.205	0.132	0.101
32000	0.507	0.556	0.568	0.425	0.256	0.136	0.106	0.097
64000	0.233	0.235	0.238	0.209	0.138	0.105	0.100	0.094
128000	0.153	0.135	0.141	0.126	0.115	0.105	0.096	0.095

Table S4. Square matrix titration of GM monoclonal antibody of ELISA

Mab dilution (1:X)	Coating antigen (μg/mL)							
	8	4	2	1	0.5	0.25	0.125	0.0625
500	2.804	2.938	2.799	2.582	1.480	0.542	0.234	0.177
1000	2.554	2.416	2.484	2.168	1.316	0.446	0.200	0.174
2000	1.996	1.906	1.823	1.610	1.057	0.357	0.196	0.156
4000	1.351	1.314	1.267	1.225	0.798	0.287	0.167	0.148
8000	0.818	0.838	0.847	0.751	0.546	0.226	0.152	0.157
16000	0.564	0.568	0.544	0.500	0.368	0.180	0.138	0.136
32000	0.375	0.376	0.378	0.345	0.262	0.158	0.137	0.135
64000	0.292	0.269	0.26	0.243	0.198	0.133	0.127	0.139

Table S5. Square matrix titration of SM2 monoclonal antibody of ELISA

Coating antigen	Mab dilution (1:X)							
(μg/mL)	5000	10000	20000	40000	80000	160000	320000	640000
32	3.108	2.814	1.214	0.483	0.204	0.128	0.138	0.118
16	3.220	2.692	1.123	0.454	0.201	0.136	0.188	0.119
8	2.933	2.115	0.795	0.348	0.180	0.132	0.140	0.117
4	2.002	1.069	0.415	0.247	0.148	0.146	0.126	0.122
2	0.591	0.334	0.178	0.154	0.152	0.148	0.173	0.171
1	0.378	0.211	0.157	0.148	0.124	0.162	0.148	0.114
0.5	0.253	0.188	0.143	0.132	0.138	0.142	0.155	0.132
0.25	0.249	0.182	0.149	0.149	0.147	0.171	0.167	0.131

Table S6. CLX antibody chip after be stored at 4°C

Time (m)	CLX (μg/L)					
	10	5	2.5	1.25	0.625	0
0	4074	6722	11770	15782	19309	26165
	4268	7024	10999	16136	21441	24442
	4444	7758	12535	17138	20932	25149
2	3125	7474	8237	11748	15732	22521
	3593	6980	8840	12134	16046	23394
	3460	7293	8967	10419	14202	21954
4	3464	5044	9076	11564	14461	18872
	3158	5988	8596	12610	15292	17072
	3260	5684	9215	11960	14314	17887
6	2999	5125	8906	10641	14903	17356
	2733	4918	8635	9527	15587	16925
	2828	4981	8386	10086	14694	16717
8	2563	4660	8329	11507	13086	15110
	2419	4514	8034	10594	13560	15278
	2390	4608	7888	10844	12953	14968

Table S7. CLX antibody chip after be stored at 37°C

Time (d)	CLX (μg/L)					
	10	5	2.5	1.25	0.625	0
0	2565	7400	12340	16544	22419	23547
	2463	6518	12021	16201	19728	24544
	2546	6836	11042	15629	21312	25232
2	3013	5954	10843	16943	14538	21336
	3212	6440	9909	17898	13749	21306
	3097	6597	9523	18948	13698	20399
4	3235	6549	8936	11612	14737	17971
	3710	6264	9135	10914	15258	17969
	3503	6301	9523	11463	14382	18738
6	3291	5757	9268	11548	13966	16647
	3104	6089	9087	11216	14583	18735
	3257	6282	9150	12113	14604	17834
8	2162	5005	8102	9707	12548	14460
	2300	4626	7626	9754	11958	14406
	2231	5074	7456	9856	11745	13953

Table S8. CRO antibody chip after be stored at 4°C

Time (m)	CET (μg/L)					
	10	5	2.5	1.25	0.625	0
0	3948	6408	12478	21224	15878	23518
	3543	8001	11783	19081	16912	24458
	3774	7922	12089	18958	15905	25906
2	2343	6960	11022	17638	21474	26724
	2426	6709	10859	16350	20124	21956
	2529	7566	12482	17418	19126	23171
4	3638	6890	10733	13612	17446	19843
	3277	7813	9984	11795	16447	20123
	3568	6353	9456	15034	15623	20932
6	2486	4904	9315	11702	14853	17658
	2825	4866	9165	11846	15234	15958
	2353	4874	10209	13542	17568	18876
8	2928	5940	7080	12000	15745	16968
	2522	5270	8697	11697	13789	15915
	2976	6089	8350	10088	13260	15190

Table S9. CRO antibody chip after be stored at 37℃

Time (d)	CET (μg/L)					
	10	5	2.5	1.25	0.625	0
0	2312	5254	8352	12079	15829	17541
	2361	6359	8921	12868	14170	18110
	2098	6030	8422	11786	13563	18930
2	1952	4297	8190	10054	12784	15883
	2120	4825	7007	9994	13222	16349
	2202	5092	8573	9780	13702	14502
4	2548	4948	7654	9582	10781	14817
	2874	4874	8007	9618	11942	14743
	2454	5054	7669	10175	12004	15050
6	2203	4033	6676	9626	11857	12872
	2551	4336	7074	8976	10767	13496
	2096	3948	6623	9655	11857	13953
8	1377	3536	5567	7596	9507	10846
	1374	3832	6073	8143	9586	9932
	1472	3616	4983	8312	10672	12140

Table S10. N antibody chip after be stored at 4℃

Time (m)	NEO (μg/L)					
	10	5	2.5	1.25	0.625	0
0	1837	3883	6261	7863	10116	12578
	2084	3768	5916	8067	9331	13518
	1951	3956	5705	7355	8859	13225
2	1622	3407	5025	6869	9563	12234
	1448	3025	4973	7493	8953	11476
	1435	2960	4663	7379	9455	11850
4	1334	2490	4768	6259	8009	10267
	1350	2600	4586	5910	8204	10149
	1180	2807	4340	6082	7572	9890
6	1257	2345	4489	6178	7544	9671
	1268	2447	4220	6140	7731	9563
	1110	2644	4186	6382	7132	9033
8	1127	2140	4148	5479	7137	8564
	1142	2262	4000	5806	7050	8685
	1265	2860	4212	5680	6598	8395

Table S11. N antibody chip after be stored at 37℃

Time (d)	NEO (μg/L)					
	10	5	2.5	1.25	0.625	0
0	1654	2602	5296	6451	8457	10639
	1492	2650	5005	6347	7893	10834
	1650	2758	4930	6222	7593	11732
2	1508	2877	4321	6087	7219	9834
	1777	2821	4500	5804	7514	10315
	1621	3093	4572	5774	7290	10197
4	1107	2278	3512	4915	6503	8475
	1035	2130	3396	5262	6475	8344
	1009	2175	3366	5152	6627	8306
6	825	1894	3225	4447	6712	7764
	936	1943	3359	4598	5817	7659
	853	2174	3365	5034	6588	7636
8	916	1720	2864	4247	5999	6485
	942	1640	3219	4368	5835	7228
	823	1564	2976	3934	5221	6490

Table S12. GM antibody chip after be stored at 4℃

Time (m)	GNE (μg/L)					
	80	40	20	10	5	0
0	900	2606	3807	5527	7497	9678
	987	2578	3692	5152	6718	8987
	946	2742	3587	5452	7005	9347
2	898	2087	3955	5016	6753	8734
	846	1987	3778	5310	6654	8423
	868	1876	3863	4909	6876	8347
4	802	2097	3679	4556	5974	7903
	747	2208	3702	4768	6082	7953
	763	2308	3450	4424	6298	7508
6	760	2044	3567	4240	5972	7381
	720	2168	3230	4505	6109	7444
	736	2253	3448	4150	5980	7254
8	648	1346	3151	3546	5042	5984
	598	1474	2640	3673	4537	5874
	684	1408	2853	3872	4873	5763

Table S13. GM antibody chip after be stored at 37°C

Time (d)	GNE (μg/L)					
	80	40	20	10	5	0
0	821	2359	3487	4907	6689	8739
	889	2110	3149	4680	6128	8540
	799	2209	3398	5003	6395	8567
2	834	2157	4084	5021	6945	8974
	849	2264	4174	4709	6563	9003
	794	2375	3843	5084	6825	8844
4	780	2408	3357	4689	6578	8275
	841	2156	3326	4894	5904	7842
	837	2304	3258	4569	6237	8060
6	578	1789	2753	4082	5032	6563
	608	1874	2903	3761	5252	6764
	634	1894	2632	3683	5347	6345
8	660	1799	2787	3732	5238	6385
	627	1804	2709	3913	4983	6423
	642	2008	2818	3609	5093	6224

Table S14. SM2 antibody chip after be stored at 4°C

Time (m)	SM2 (μg/L)					
	24	12	6	3	1.5	0
0	1197	4478	7587	11354	14875	18632
	1308	4269	7692	11992	13697	18132
	1264	4109	7348	10737	13984	17553
2	1509	4147	7566	11087	12983	17383
	1371	3951	7342	10839	14420	16939
	1405	3964	7565	10716	13392	17326
4	1208	4071	7087	10245	13045	16578
	1193	3953	7001	10664	13675	16206
	1305	3814	7592	10332	14082	16951
6	1523	3982	6823	10584	11693	15253
	1423	3687	6670	9897	13839	15531
	1398	3598	6447	10560	12396	15946
8	1008	3590	6692	8382	11809	13956
	974	3929	6866	8501	12232	14200
	1045	3625	6501	8097	13087	13940

Table S15. SM2 antibody chip after be stored at 37℃

Time (m)	SM2 (μg/L)					
	24	12	6	3	1.5	0
0	1497	3456	6521	9990	12093	16239
	1633	3513	6625	10087	11580	17093
	1568	3582	6783	10038	12708	17003
2	1542	4221	6573	9393	11237	16094
	1634	4414	6032	9573	12280	15239
	1783	4067	6397	9087	10807	15994
4	1498	3228	5897	8093	9945	14075
	1545	2999	5715	8293	10747	13905
	1408	3185	6003	7933	9942	12940
6	960	3108	5308	8078	10053	13094
	967	3208	5349	8398	9805	12790
	1018	3009	5759	8175	10738	13489
8	1264	3207	5307	8563	10032	12582
	1204	3016	5452	8047	9792	13192
	1180	2900	5053	8035	9445	11998

Table S16. Recoveries of CLX and similar drugs at three levels in milk

Drugs	Spiked concentration (μg/kg)	Recovery (%) ($\overline{X}$ ±SD)	$CV_{intra-assay}$ (%，n=3)	Recovery (%) ($\overline{X}$ ±SD)	$CV_{inter-assay}$ (%，n=9)
CLX	50	84.63±1.70	2.0	87.78±6.43	7.3
		83.53±6.97	8.3		
		95.17±2.82	5.2		
	100	102.22±13.18	12.9	100.42±9.03	9.0
		108.42±4.13	3.8		
		90.63±5.27	5.8		
	200	113.62±5.87	5.6	103.33±9.98	9.7
		93.69±9.52	10.2		
		102.69±4.15	4.6		
CFR	50	89.34±7.97	8.9	88.65±4.47	5.0
		92.74±9.68	10.4		
		83.88±6.76	8.1		
	100	103.13±4.24	4.1	93.98±7.93	8.4
		89.60±4.14	4.6		
		89.19±3.57	4.0		
	200	95.23±4.77	5.0	89.58±5.62	6.3
		83.99±2.7	3.2		
		89.53±5.92	6.6		
CE	50	111.36±7.32	6.6	105.29±6.01	5.7
		99.34±11.56	11.6		
		105.18±4.85	4.6		
	100	108.45±2.56	2.4	103.10±6.99	6.8
		95.19±3.62	3.8		
		105.66±5.77	5.5		
	200	104.83±9.81	9.4	96.67±8.15	8.4
		96.64±9.72	10.1		
		88.54±5.16	5.8		

Table S17. Recoveries of CLX and similar drugs at three levels in pork

Drugs	Spiked concentration (μg/kg)	Recovery (%) ($\overline{X}$ ±SD)	$CV_{intra-assay}$ (%，n=3)	Recovery (%) ($\overline{X}$ ±SD)	$CV_{inter-assay}$ (%，n=9)
CLX	100	87.83±5.27	5.6	96.51±7.66	7.9
		102.31±9.63	9.4		
		99.39±7.64.	7.7		
	200	104.96±5.02	4.8	96.54±8.34	8.6
		96.37±9.16	9.5		
		88.28±6.90	7.8		
	400	91.20±5.08	5.6	96.69.±5.96	6.2
		103.30±9.97	9.7		
		95.83±3.64	3.6		
CFR	100	101.84±3.95	3.9	94.23±7.16	7.6
		87.62±6.67	7.6		
		93.23±5.32	5.7		
	200	99.05±5.39	5.4	95.06±3.59	3.8
		92.11±5.39	5.9		
		94.02±3.43	3.7		
	400	98.71±3.13	3.2	92.42±5.86	6.3
		87.11±3.12	3.6		
		91.43±2.45	2.7		
CE	100	88.89±2.86	3.2	95.40±8.13	8.5
		104.52±7.80	7.5		
		92.79±5.80	6.3		
	200	98.14±8.13	8.3	91.85±6.09	6.6
		91.41±8.38	9.2		
		85.99±2.83	3.3		
	400	93.21±4.12	4.4	87.78±4.81	5.5
		86.06±5.53	6.4		
		84.07±3.53	4.2		

Table S18. LODs of CLX and similar drugs in samples

Drugs	Matrix	20 blank samples	Standard deviation	LOD	LOQ
		Measured value (μg/kg)	(SD)	(μg/kg)	(μg/kg)
CLX	Milk	18.08	0.99	21.0	27.9
	Pork	18.02	1.08	21.3	28.8
CFR	Milk	16.12	1.18	19.7	27.9
	Pork	15.84	1.05	19.0	26.3
CE	Milk	17.60	1.06	20.8	28.3
	Pork	16.26	0.97	19.2	26.0

Table S19. Recoveries of CRO and similar drugs at three levels in milk

Drugs	Spiked concentration (μg/kg)	Recovery (%) ($\overline{X}$ ±SD)	$CV_{intra\text{-}assay}$ (%，n=3)	Recovery (%) ($\overline{X}$ ±SD)	$CV_{inter\text{-}assay}$ (%，n=9)
CRO	50	99.04±9.27	9.4	91.77±6.82	7.4
		90.77±2.68	3.0		
		85.51±3.45	4.0		
	100	81.56±5.15	6.3	85.92.±4.44	5.2
		85.70±2.50	2.9		
		90.47±9.36	10.4		
	200	108.74±4.47	4.1	105.96±8.73	8.2
		112.96±4.41	3.9		
		96.18±5.80	6.0		
EFT	50	89.53±4.34	4.8	94.41±7.77	8.2
		90.33±3.63	4.0		
		103.37±7.74	7.5		
	100	109.98±6.28	5.7	101.16±9.41	9.3
		102.26±9.41	9.2		
		91.25±7.98	8.7		
	200	95.95±5.11	5.3	96.59±6.06	6.3
		90.87±5.35	5.9		
		102.94±4.6	4.5		
DFC	50	98.56±5.78	5.9	99.66±6.80	6.8
		106.94±6.63	6.2		
		93.48±5.22	5.6		
	100	88.90±3.88	4.4	97.48±8.35	8.6
		105.59±9.21	8.7		
		97.94±9.45	9.7		
	200	98.56±3.86	3.9	98.03±6.07	6.2
		91.72±5.90	6.4		
		103.81±5.19	5.0		

Table S20. Recoveries of CRO and similar drugs at three levels in pork

Drugs	Spiked concentration (μg/kg)	Recovery (%) ($\bar{X}$ ±SD)	$CV_{intra-assay}$ (%，n=3)	Recovery (%) ($\bar{X}$ ±SD)	$CV_{inter-assay}$ (%，n=9)
CRO	100	105.52±5.77	5.5	97.26±7.15	7.4
		93.32±8.13	8.7		
		92.95±3.52	3.8		
	200	83.62±1.34	1.6	87.89±4.38	5.0
		92.40±5.90	6.4		
		87.67±7.85	9.0		
	400	97.64±7.52	7.7	91.83±5.21	5.7
		90.27±6.4	7.1		
		87.57±2.77	3.2		
EFT	50	89.53±4.34	4.8	94.41±7.77	8.2
		90.33±3.63	4.0		
		103.37±7.74	7.5		
	100	109.98±6.28	5.7	101.16±9.41	9.3
		102.26±9.41	9.2		
		91.25±7.98	8.7		
	200	95.95±5.11	5.3	96.59±6.06	6.3
		90.87±5.35	5.9		
		102.94±4.6	4.5		
DFC	100	96.06±4.84	5.0	89.36±6.2	6.9
		83.82±4.47	5.3		
		88.20±4.11	4.7		
	200	87.53±5.76	6.6	88.99±4.59	5.2
		85.30±5.18	6.1		
		94.13±5.76	6.1		
	400	91.59±3.63	4.0	90.77±3.82	4.2
		94.12±6.27	6.7		
		86.61±4.65	5.4		

Table S21. LODs of CRO and similar drugs in samples

Drugs	Matrix	20 blank samples Measured value (μg/kg)	Standard deviation (SD)	LOD (μg/kg)	LOQ (μg/kg)
CRO	Milk	17.86	1.33	21.9	31.2
	Pork	15.83	1.10	19.1	26.8
EFT	Milk	16.62	1.18	20.2	28.4
	Pork	14.80	1.02	17.9	25.0
DFC	Milk	17.28	1.15	20.7	28.8
	Pork	17.41	1.08	20.6	28.2

Table S22. Recoveries of N and similar drugs at three levels in milk

Drugs	Spiked concentration (μg/kg)	Recovery (%) ($\bar{X}$ ±SD)	$CV_{intra-assay}$ (%，n=3)	Recovery (%) ($\bar{X}$ ±SD)	$CV_{inter-assay}$ (%，n=9)
N	75	84.46±3.72	4.4	92.69±8.16	8.8
		92.82±9.57	10.3		
		100.78±7.92	7.9		
	150	84.23±6.79	8.1	90.26±7.06	7.8
		88.52±5.78	6.5		
		98.02±3.61	3.7		
	300	96.36±8.01	8.3	104.33±7.14	6.9
		110.15±7.31	6.6		
		106.48±9.21	8.7		
AK	75	91.00±6.22	6.8	96.16±6.21	6.5
		103.05±9.23	9.0		
		94.43±7.18	7.6		
	150	110.98±4.01	3.6	104.73±7.68	7.3
		96.16±4.05	4.2		
		107.05±3.81	3.6		
	300	85.94±2.97	3.5	90.86±4.44	4.9
		92.10±3.14	3.4		
		94.55±8.99	9.5		
PRM	75	92.83±8.05	8.7	101.43±7.54	7.4
		104.55±7.88	7.5		
		106.92±3.3	3.1		
	150	96.20±4.49	4.7	96.45±7.74	8.0
		104.31±0.77	0.7		
		88.83±2.29	2.6		
	300	86.65±1.85	2.1	91.26±4.01	4.4
		93.84±3.54	3.8		
		93.30±4.18	4.5		

Table S23. Recoveries of N and similar drugs at three levels in pork

Drugs	Spiked concentration (μg/kg)	Recovery (%) ($\overline{X}$ ±SD)	$CV_{intra-assay}$ (%，n=3)	Recovery (%) ($\overline{X}$ ±SD)	$CV_{inter-assay}$ (%，n=9)
N	75	84.59±4.46	5.3	89.2±4.92	5.5
		88.63±3.64	4.1		
		94.37±1.86	2.0		
	150	87.61±6.98	8.0	92.97±5.42	5.8
		98.44±5.13	5.2		
		92.86±7.15	7.7		
	300	96.54±2.57	2.7	95.2±3.92	4.1
		98.29±6.13	6.2		
		90.79±2.60	2.9		
AK	75	94.53±7.03	7.4	90.63±3.70	4.1
		87.17±7.91	9.1		
		90.18±3.23	3.6		
	150	92.73±6.49	7.0	93.47±7.65	8.2
		86.22±1.11	1.3		
		101.47±3.28	3.2		
	300	107.36±3.31	3.1	97.52±8.98	9.2
		95.41±4.91	5.2		
		89.78±3.73	4.2		
PRM	75	99.42±10.91	11.0	97.29±7.06	7.3
		89.40±10.66	11.9		
		103.04±9.25	9.0		
	150	86.03±2.94	3.4	92.93±6.03	6.5
		97.16±8.32	8.6		
		95.61±3.99	4.2		
	300	90.82±4.33	4.8	93.09±6.36	6.8
		100.27±3.54	3.5		
		86.61±4.65	5.4		

Table S24. LODs of N and similar drugs in samples

Drugs	Matrix	20 blank samples Measured value (μg/kg)	Standard deviation (SD)	LOD (μg/kg)	LOQ (μg/kg)
N	Milk	16.61	0.79	19.0	24.5
	Pork	16.24	0.47	17.6	20.9
AK	Milk	21.04	0.97	23.9	30.7
	Pork	20.85	0.90	23.6	29.9
PRM	Milk	11.00	0.75	13.2	18.5
	Pork	10.80	0.69	12.9	17.7

Table S25. Recoveries of GM at three levels in milk and pork

Drugs	Spiked concentration (μg/kg)	Recovery (%) ($\bar{X}$ ±SD)	$CV_{intra\text{-}assay}$ (%，n=3)	Recovery (%) ($\bar{X}$ ±SD)	$CV_{inter\text{-}assay}$ (%，n=9)
Milk	50	109.27±5.69	5.2	100.56±8.58	8.5
		92.13±8.83	9.6		
		100.28±10.4	10.4		
	100	100.70±9.33	9.3	94.48±7.71	8.2
		85.85±6.16	7.2		
		96.89±4.34	4.5		
	200	94.12±6.21	6.6	93.42±7.24	7.8
		100.28±6.14	6.1		
		85.86±2.28	2.7		
Pork	50	110.15±5.09	4.6	102.79±8.49	8.3
		104.73±3.83	3.7		
		93.50±6.19	6.6		
	100	102.35±6.8	6.7	92.74±8.42	9.1
		89.2±8.24	9.2		
		86.67±5.43	6.3		
	200	89.35±5.04	5.6	95.49±6.01	6.3
	50	101.36±5.11	5.0		
		95.77±5.19	5.4		

Table S26. LODs of GM in samples

Drugs	Matrix	20 blank samples Measured value (μg/kg)	Standard deviation (SD)	LOD (μg/kg)	LOQ (μg/kg)
GM	Milk	12.28	1.04	15.4	22.7
	Pork	12.37	0.91	15.1	21.5

Table S27. Recoveries of Sulfonamides at three levels in milk

Drugs	Spiked concentration (μg/kg)	Recovery (%) ($\bar{X}$ ±SD)	$CV_{intra-assay}$ (%，n=3)	Recovery (%) ($\bar{X}$ ±SD)	$CV_{inter-assay}$ (%，n=9)
SM2	50	97.35±4.33	4.5	95.31±6.07	6.4
		100.10±5.86	5.9		
		88.48±4.84	5.5		
	100	96.55±8.00	8.3	105.31±7.69	7.3
		110.97±6.59	5.9		
		108.41±5.61	5.2		
	200	96.34±9.76	10.1	98.67±4.63	4.7
		103.99±9.42	9.1		
		95.66±4.94	5.2		
SD	50	97.82±10.57	10.8	95.85±6.51	6.8
		101.15±5.25	5.2		
		88.58±2.37	2.7		
	100	102.61±5.49	5.4	98.32±6.26	6.4
		91.14±9.89	10.9		
		101.21±3.44	3.4		
	200	95.16±4.72	5.0	90.84±4.61	5.1
		91.39±5.16	5.7		
		85.98±4.09	4.8		
SDM	50	92.82±6.48	7.0	94.39±4.63	4.9
		99.6±6.32	6.3		
		90.75±3.36	3.7		
	100	93.19±9.47	10.2	95.85±9.73	10.2
		87.73±4.76	5.4		
		106.63±5.63	5.3		
	200	107.11±4.25	4.0	98.25±8.29	8.4
		96.95±4.97	5.1		
		90.68±5.75	6.3		
SM1	50	102.16±5.93	5.8	97.48±4.81	4.9

		92.55±7.32	7.9		
		97.73±12.60	12.9		
	100	89.82±7.30	8.1	94.62±4.47	4.7
		95.41±8.66	9.1		
		98.65±9.75	9.9		
	200	95.58±8.93	9.4	102.53±9.29	9.1
		98.91±8.43	8.5		
		113.08±4.23	3.7		
SPD	50	102.24±8.81	8.6	96.86±8.62	8.9
		101.43±9.57	9.4		
		86.92±3.88	4.5		
	100	99.2±8.06	8.1	95.39±3.33	3.5
		93.9±9.07	9.7		
		93.06±9.50	10.2		
	200	107.63±2.00	1.9	95.37±11.07	11.6
		92.35±3.75	4.1		
		86.12±3.09	3.6		
SPZ	50	91.39±7.32	8.0	99.01±6.66	6.7
		101.99±8.62	8.5		
		103.67±6.89	6.7		
	100	96.55±8.00	8.3	105.31±7.69	7.3
		110.97±6.59	5.9		
		108.41±5.61	5.2		
	200	101.37±7.93	7.8	92.81±7.89	8.5
		91.22±5.40	5.9		
		85.83±2.26	2.6		

Table S28. Recoveries of Sulfonamides at three levels in pork

Drugs	Spiked concentration (μg/kg)	Recovery (%) ($\overline{X}$ ±SD)	$CV_{intra-assay}$ (%，n=3)	Recovery (%) ($\overline{X}$ ±SD)	$CV_{inter-assay}$ (%，n=9)
SM2	50	103.72±5.64	5.4	98.33±5.22	5.3
		98.00±9.77	10.0		
		93.29±5.94	6.4		
	100	91.31±9.45	10.4	94.11±9.32	9.9
		86.52±2.82	3.3		
		104.51±8.92	8.5		
	200	101.94±7.24	7.1	97.81±7.01	7.2
		101.78±7.52	7.4		
		89.71±4.99	5.6		

SD	50	92.58±6.22	6.7	96.47±4.39	4.6
		95.59±9.16	9.6		
		101.23±9.40	9.3		
	100	84.45±5.95	7.1	88.32±7.34	8.3
		96.78±6.99	7.2		
		83.73±5.58	6.7		
	200	87.60±3.90	4.5	93.01±4.71	5.1
		95.21±5.80	6.1		
		96.21±5.30	5.5		
SDM	50	100.39±9.48	9.4	99.92±8.53	8.5
		108.21±4.85	4.5		
		91.16±4.10	4.5		
	100	88.02±7.43	8.4	89.86±6.66	7.4
		97.25±9.40	9.7		
		84.32±4.69	5.6		
	200	89.63±5.60	6.2	99.11±8.93	9.0
		107.37±7.90	7.4		
		100.33±5.86	5.8		
SM1	50	88.22±8.13	9.2	93.52±6.12	6.5
		92.11±8.75	9.5		
		100.21±4.79	4.8		
	100	93.82±8.45	9.0	90.55±2.88	3.2
		88.36±5.04	5.7		
		89.48±6.93	7.7		
	200	90.76±5.00	5.5	93.41±4.48	4.8
		98.58±5.42	5.5		
		90.9±8.97	9.9		
SPD	50	92.58±6.22	6.7	99.07±6.37	6.4
		105.31±8.31	7.9		
		99.32±10.08	10.2		
	100	95.49±7.01	7.4	94.69±5.99	6.3
		100.24±3.93	3.9		
		88.34±8.72	9.9		
	200	88.69±1.01	1.1	93.68±4.50	4.8
		97.46±4.23	4.3		
		94.90±6.33	6.7		
SPZ	50	99.14±8.98	9.1	96.47±4.31	4.5
		98.77±6.87	7.0		
		91.50±9.57	10.5		
	100	106.69±9.69	9.1	102.44±4.63	4.5
		97.51±4.71	4.8		

	103.14±8.87	8.6		
200	86.43±3.32	3.8	93.72±6.35	6.8
	96.75±3.04	3.1		
	97.99±5.91	6.0		

Table S29. LODs of Sulfonamides in samples

Drugs	Matrix	20 blank samples Measured value (μg/kg)	Standard deviation (SD)	LOD (μg/kg)	LOQ (μg/kg)
SM2	Milk	16.62	1.45	21.0	31.1
	Pork	15.88	1.17	19.4	27.6
SD	Milk	15.21	0.80	17.6	23.2
	Pork	16.07	1.03	19.2	26.4
SDM	Milk	14.98	0.92	17.7	24.2
	Pork	16.98	1.14	20.4	28.4
SM1	Milk	16.17	0.98	19.1	26.0
	Pork	15.87	1.07	19.1	26.6
SPD	Milk	15.34	0.68	17.4	22.1
	Pork	16.45	0.53	18.0	21.8
SPZ	Milk	14.87	1.06	18.1	25.5
	Pork	15.71	0.82	18.2	23.9
